# State Registration at Hand

**Published:** 1919-04-05

**Authors:** 


### THE MATRONS' AND SISTERS' DEPARTMENT.

### STATE REGISTRATION AT HAND.

The Bill for the State Registration of Nurses, put forward by the Central Committee, met with an exceedingly cordial reception in the House of Commons on its second reading on March 28. Some admirable speeches heralded its appearance. Opposition was conspicuous by its absence for Mr. Rawlinson of Cambridge University, who expressed his dislike to the principle of registration, did not vote against the Bill. Sir Samuel Scott, who appealed to the Local Government Board for support, declared that the promotors of the Bill were ready to assent to any reasonable amendments, and Major Astor, Parliamentary Secretary to the Local Government Board, pronounced himself in favour of the principle of State Registration for Nurses. All this is much, very much. It marks a miraculous change in the aspect under which the subject of State Registration has hitherto been surveyed. The College of Nursing during the short three years of its existence has completely brought over informed nursing opinion, so long indifferent or even hostile to the principle of registration, to accept its general principles, and the result is seen in the attitude of the House of Commons. So long as a large majority of nurses opposed registration the fate of any bill designed to it was a foregone conclusion. Once bring nurses in a body to accept its principles and it was certain that Parliament would readily assent to their desires. The tragedy of the Central Committee's long endeavours has lain in their inability to secure the confidence of nurses in their proposed form of legislation.

Having been read a second time the Bill passes to a Standing Committee of the House in order to be examined, criticised and amended. Its fate partly depends on the statesmanlike qualities exhibited at this stage by its promoters. Major Astor stated that another Bill for the Registration of Nurses had been prepared and that the points of difference between the two Bills would be considered in Committee. The attitude which the Government would take up towards the Bill after it passed through Committee would entirely depend on the shape in which it returned to the House. He welcomed any measure which would improve the status of a profession that was the admiration of the world, and which had during the war earned the undying gratitude of millions.

These utterances make it clear that the atmosphere of suspicion and distrust which has so long hovered over the supporters of the present Bill must be changed if the Bill is to become law. Conciliation has long been the policy of the College. Lt.-Colonel Raw, who has the College Bill in charge, warmly supported the principle of registration contained in the Bill now before Parliament. Sir Arthur Stanley in a letter published in our last issue stated that the Council of the College of Nursing has decided that as the Bill affirms the principle of State recognition of the nursing profession it should so far be supported.

What more then is needed? A little forbearance, a spirit of give-and-take, a removal of the whole subject out of the heated stages of controversy into a calm consideration of its bearing on the welfare of nurses and the protection of the public. Undoubtedly this public-spirited attitude will be adopted towards the whole matter in committee. We rejoice to hope that points of administration and control should be examined and decided by members sitting in conclave unbiased by long buried controversies and solely moved by considerations of the best form of organisation which can be devised to carry out the principles of State Registration.

The nursing profession is passing through a period of rapid development and change. We hold that it is essential that the provisions laid down for its regulation by Parliament should not preclude alterations in the future. Under the College Bill its regulations may be changed or modified under the authority of the Privy Council after being laid before both Houses of Parliament, and thus legislation would not retard progress. The Bill now before the House cannot be modified in the smallest particular without the sanction of an Amending Act. In its faulty provisions for representation on the General Nursing Council and in the undue powers accorded to the Provisional Council the present Bill is also very defective. There will be keen suspense as to the result of the deliberations which members of the House are devoting to these important points.

